# Predicting bowel necrosis in pediatric acute intussusception using roundness and other related factors

**DOI:** 10.1186/s12887-025-06172-9

**Published:** 2025-10-21

**Authors:** Xin-Xin Xu, Yi-Jin Cai, Jia Liu, Yi Zhuang, Qi Ma

**Affiliations:** 1 Department of Ultrasound, Affiliated Children’s Hospital of Jiangnan University（Wuxi Children’s Hospital, Wuxi, 214023 Jiangsu China; 2 Department of General Surgery, Affiliated Children’s Hospital of Jiangnan University（Wuxi Children’s Hospital), Wuxi, 214023 Jiangsu China; 3 Department of Orthopedic Surgery, Changzheng Hospital, Naval Medical University, Shanghai, 200003 P.R. China; 4 Department of Diagnostic Radiology, Affiliated Children’s Hospital of Jiangnan University (Wuxi Children’s Hospital), Wuxi, Jiangsu 214023 China; 5 Department of Ultrasound, The Second Affiliated Hospital of Soochow University (The General Hospital of Nuclear Industry), Suzhou, 215004 Jiangsu China

**Keywords:** Intussusception, Ultrasonography, Bowel necrosis

## Abstract

**Objective:**

This study aimed to investigate the risk factors of intestinal necrosis in children with intussusception and intestinal necrosis was established.

**Methods:**

The clinical data of children diagnosed with intestinal necrosis after surgical treatment in our hospital were retrospectively analyzed and assigned to the bowel necrosis group.A control group was established treated successfully with air enema, without bowel necrosis, during the same period. Ultrasonic manifestation and clinical features were recorded and analyzed. Factors associated with bowel necrosis were analyzed using univariate and multivariate unconditional logistic regression analyses.

**Results:**

(1) The bowel necrosis group included a higher proportion of children under 12 months of age, and had more cases with blood flow signal < grade 4, peritoneal effusion, and bloody stools (*P* < 0.05) than the non-intestinal necrosis group. The values for roundness, concentric ring thickness, length of the intussuscepted segment, head-to-neck diameter ratio, bowel wall thickness, and neutrophil-to-lymphocyte ratio ( NLR) were all higher in the bowel necrosis group (*P* < 0.05). (2) The logistic regression analysis indicated that roundness (×100) [odds ratio (*OR*) = 1.397, 95% confidence interval (*CI*): 1.086–1.796] and blood flow signal (< grade 4) (*OR* = 0.099, 95% *CI*: 0.018–0.543) were independent predictors of bowel necrosis in intussusception.

**Conclusions:**

Roundness and blood flow signal grading are independent predictors for diagnosing bowel necrosis in pediatric intussusception.

**Supplementary Information:**

The online version contains supplementary material available at 10.1186/s12887-025-06172-9.

## Introduction

Intussusception is a common condition in infants and young children, characterized by the invagination of a segment of the intestine and its mesentery into an adjacent intestinal lumen, leading to bowel obstruction [1, 2]. Also, 80–90% of intussusception cases are reported in children aged less than 2 years [3].Intussusception can be classified into simple and complex types; complex intussusception refers to a previously intussuscepted segment re-invaginating into another adjacent intestinal segment.

The primary treatment method for intussusception is air enema. Kaplan et al. [4] reported a 92% success rate with air enema reduction, although some children require surgery due to unsuccessful enema reduction. Bowel necrosis is a severe complication of intussusception; however, early symptoms are often nonspecific, and routine blood tests, biochemical assays, abdominal radiography, and computed tomography often lack sensitivity in diagnosing bowel necrosis [5, 6]. Ultrasound is primarily used to diagnose intussusception. However, studies suggest that it may also have value in predicting bowel necrosis. Hanquinet [7] conducted research and discovered that ultrasound exhibited different manifestations in two cases of similar irreducible intestinal intussusception accompanied by intestinal wall necrosis and perforation. Color Doppler ultrasound showed that there was blood flow within the intestinal tube in one case of intussusception, while there was no blood flow in the other case.He analyzed the predictive value of Doppler ultrasound in diagnosing the viability of intestinal tissues.Chen et al. [8] observed that the bowel wall of the invaginated segment was obviously thickened and the center of the invaginated segment was often accompanied with swollen lymph node and appendix caecalis, and the intussusceptional fluidify, the expanding of distal segment accompanied with the thickened bowels wall, and weakening or disappearance of enterokinesia were the appearances of necrosis of most of bowel walls. Huang et al. [9] found that complex intussusception more likely led to bowel necrosis due to increased tension and mesenteric vascular compression. These studies suggested that the greater the content volume in the intussuscepted segment, the higher the risk of severe vascular compression in the bowel wall, potentially resulting in necrosis. However, whether there is a specific parameter that can help better predict acute intussusception intestinal necrosis remains unclear.

Bae et al. [10] were the first to introduce roundness as a parameter in Magnetic resonance imaging studies of breast cancer, exploring its correlation with tumor biomarkers and subtypes. The roundness metric, ranging from 1 to 100%, gauges the degree of circularity, with values closer to 100% indicating a more circular shape. In intussusception, the cross-section often displays a “concentric ring” sign, which can range from elliptical to nearly circular depending on the volume of intussuscepted content. Thus, roundness may directly reflect the tension within the intussuscepted segment and indirectly indicate mesenteric vascular compression. The question is whether roundness, as a parameter, can be applied to the ultrasonographic characteristics of intussusception to examine the association between roundness and bowel necrosis, potentially aiding in prognostication. This study aimed to retrospectively analyze clinical data and ultrasound characteristics of children with intussusception suspected of bowel necrosis, confirmed through surgery, to develop a predictive model for bowel necrosis and assess its utility in forecasting the prognosis of intussusception.

## Materials and methods

### Clinical data

Approval for this study was obtained from the institutional review board of the Children’s Hospital of Jiangnan University.This study included 23 cases bowel necrosis in children was admitted to the Affiliated Children’s Hospital of Jiangnan University from June 2018 to December 2021, confirmed by surgical pathology, which formed the bowel necrosis group. Additionally, a control group comprised 64 cases with successful initial air enema reduction without necrosis within the same timeframe. All patients initially underwent ultrasound examination, displaying concentric ring signs and mixed echo masses, which were confirmed through air enema or surgical pathology. The clinical data recorded included age (categorized as < 12, 12–24, and > 24 months), gender, disease course, clinical symptoms (e.g., abdominal pain or crying, vomiting, bloody stools), location of the intussusception head, length of the intussuscepted segment, concentric ring width and thickness, maximum intussusception head and neck diameters, head-to-neck diameter ratio, maximum intussusception head to concentric ring thickness diameter ratio, fat core thickness, bowel wall thickness, presence of peritoneal effusion, presence of mesenteric lymphadenopathy, pretreatment C-reactive protein (CRP), neutrophil-to-lymphocyte ratio (NLR), blood flow signal grading (0–4), and roundness.

Inclusion criteria for pediatric intussusception [2]: (1) age less than 18 years; (2) symptoms such as episodic abdominal pain, vomiting, abdominal palpation of a “sausage-like” mass with firmness and tenderness, and rectal examination revealing jelly-like bloody mucus, with one or more the following clinical manifestations; (3) abdominal radiographs revealing an intestinal shadow; (4) grayscale ultrasound features: transverse cross-section of the intussusception presenting as a “concentric ring” or “target sign,” and a longitudinal view as a “pseudo-kidney” or “sandwich sign.” (5) The above (1) (2) (3) (4) conditions must be met at the same time.

Inclusion criteria for the bowel necrosis group [9]: (1) surgically confirmed bowel necrosis; (2) complete clinical data and auxiliary examinations, including white blood cell count, CRP, and ultrasound imaging.

Exclusion criteria for the bowel necrosis group: (1) Successfully restored through surgical treatment and no intestinal wall necrosis was found; (2) Incomplete medical history information.

Inclusion criteria for the control group: (1) initial successful air enema reduction, with no bowel necrosis; (2) complete clinical data and laboratory and imaging records.

Exclusion criteria [11]: (1) previous abdominal surgery; (2) self-reduced intussusception without air enema; (3) intestinal obstruction from other causes, such as adhesions, volvulus, or nonmechanical obstructions; (4) incomplete clinical data.

### Ultrasound examination

An ultrasound diagnostic system (Philips IU22) was used, equipped with an L12-5 linear array probe (5–12 MHz) and a C5-2 convex array probe (2–5 MHz). Patients were positioned supine and kept calm. First, the convex probe was used to perform a complete abdominal and pelvic scan, covering areas from the diaphragm to the pubic symphysis, scanning the intestinal tract sequentially in the lower right abdomen, upper right abdomen, upper left abdomen, lower left abdomen, and mid-abdomen to locate the intussusception lesion.

The parameters measured included the location of the intussusception head, length of the intussuscepted segment, concentric ring width and thickness, ratio of concentric ring thickness to width, maximum intussusception head and neck diameters, head-to-neck diameter ratio, maximum intussusception head to concentric ring thickness diameter ratio, fat core thickness, bowel wall thickness, peritoneal effusion, and mesenteric lymphadenopathy. The diagnostic criteria for lymphadenopathy were as follows [12]: presence of more than one lymph node within the mesentery with a short diameter > 0.5 cm and one of the following: (a) long diameter > 1.0 cm, (b) long-to-short diameter ratio > 2, or (c) clustered arrangement with increased blood flow on color Doppler ultrasound.

**Fig. 1:**
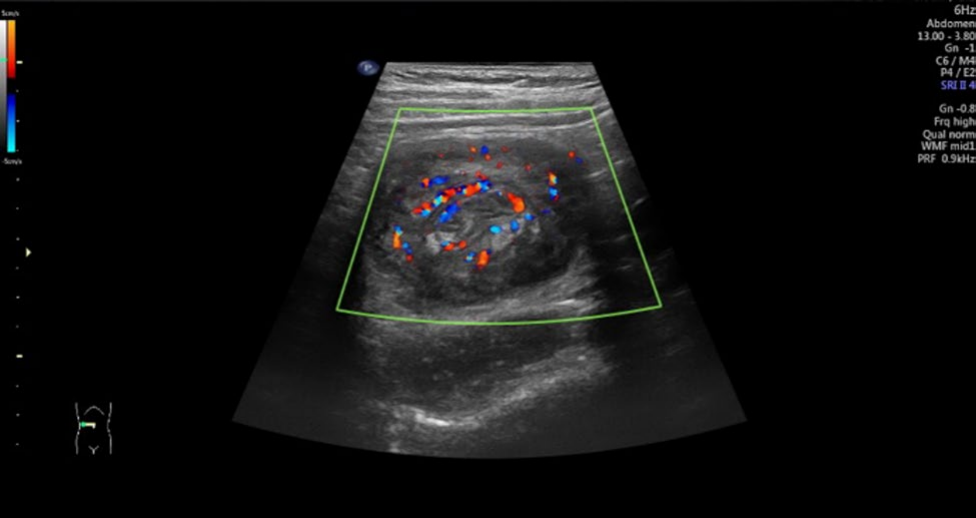
Intussusception Color Doppler Flow Signal (Grade 2)

The maximum transverse section of the intussusception neck was imaged using a linear array probe. Color Doppler flow imaging (CDFI) was set to a scale of 5 cm/s, and the color gain was increased to the threshold just below the appearance of noise. Blood flow signal grading of the intussuscepted segment was performed using the semi-quantitative Limberg method [13], with the following classification: (1) Grade 1: no abnormal blood flow detected; (2) Grade 2: spot or short-strip blood flow signals; (3) Grade 3: long-strip (> 1 cm) blood flow signals; and (4) Grade 4: long-strip blood flow signals connected to mesenteric vessels (Fig. 1).

**Fig. 2:**
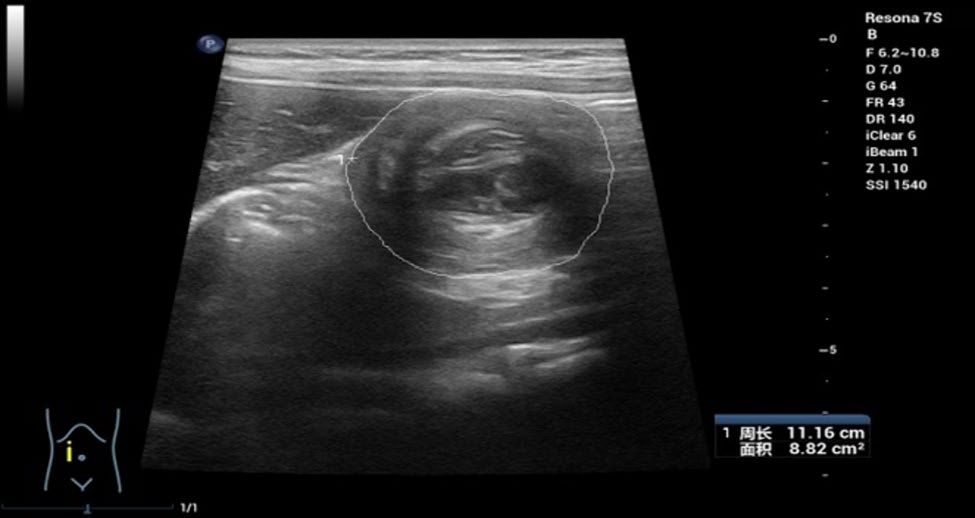
Roundness measurement diagram.Male, 11 months old. The maximum transverse area of the intussusception lesion was 8.82cm^2^, and the circumference was 11.16cm. The white ring represents the traced intussusception outline

The maximum transverse section of the intussusception was traced using the linear probe to calculate its area (*A*) and perimeter (*P*), with roundness determined using the formula [10, 14]: roundness = 4*π*× *A*/*P*^2^ (Fig. 2).

## Biochemical analysis

A CRP automatic blood analyzer (Mindray BC-7500) was used for pretreatment biochemical analysis. Five milliliters of venous blood were drawn within 2 h before surgery, allowed to stand at room temperature for 0.5–1 h, and then processed to determine CRP and NLR values.

## Treatment methods

Air enema reduction: Air (20–25 mL) was introduced through the anus under fluoroscopic guidance using an R302 MLP/A digital gastrointestinal machine (American GMM Corporation). The device was connected to a remote-controlled enema Machine, with initial air pressure at 7kPa, adjustable up to 13 kPa, to retract the intussusception head. Fluoroscopy was used to monitor the process, confirming reduction when the intussusception shadow disappeared and the small intestine expanded rapidly with gas [15]. The air enema procedures in this study were performed by senior radiologists with more than 10 years of experience.

Surgical intervention: The indications for surgery [16] included the following: (1) multiple unsuccessful air enema reductions; (2) severe bloody stools; (3) poor overall condition of the child; and (4) symptoms persisting more than 24 h with suspected bowel necrosis. The surgical approach (e.g., laparoscopic-assisted intussusception reduction or bowel resection and anastomosis) was determined based on intraoperative findings, followed by routine postoperative pharmacotherapy.

### Statistical analysis

Univariate and multivariate analyses were conducted on factors associated with the control and necrosis groups. For univariate analysis, independent-sample *t*-test was used for normally distributed continuous variables, whereas nonparametric Wilcoxon rank-sum tests were used for non-normally distributed or skewed data. Chi-square tests, Fisher’s exact test, or Wilcoxon rank-sum tests were employed for categorical variables. Multivariate analysis was conducted using binary logistic regression to construct prediction models for bowel necrosis in pediatric acute intussusception. The area under the ROC curve (AUC) was used to evaluate model discrimination, and calibration was assessed with the Hosmer–Lemeshow goodness-of-fit test. A *P-*value of < 0.05 indicated a statistically significant difference. The calculation of the sample size was estimated using the PASS2021 software.

## Results

### Univariate analysis of clinical data in the bowel necrosis group

This study was performed on 23 cases of surgically confirmed bowel necrosis, including 13 cases of primary intussusception, 4 of which were complex/compound intussusceptions and the rest were secondary intussusceptions. Further, eight primary and four secondary intussusception cases were reported in patients aged < 12 months, three primary cases in patients aged 12–24 months, and two primary and six secondary cases in patients aged > 24 months. Among these, 16 cases were lymph node intussusception or appendix compression.

Comparisons between the bowel necrosis and control groups revealed statistically significant differences in multiple factors (Tables 1, 2 and 3). The necrosis group showed higher proportion of children aged < 12 months (*χ*² = 10.442, *P* = 0.005), more cases with bloody stools (*χ*² = 10.649, *P* = 0.011), longer intussuscepted segment length (*χ*² = 521.000, *P* = 0.038), greater concentric ring thickness (*χ*² = 431.500, *P* = 0.003), a larger head-to-neck diameter ratio (*χ*² = 485.500, *P* = 0.016), thicker bowel walls (*χ*² = 482.500, *P* = 0.014), more cases with peritoneal effusion (*χ*² = 6.412, *P* = 0.011), a higher NLR (*χ*² = 398.500, *P* = 0.001), lower blood flow signal grading (*χ*² = 20.407, *P* < 0.001), and greater roundness values (*χ*² = 380.000, *P* = 0.001). Other variables showed no significant differences (*P* > 0.05). Further analysis of age groups indicated a statistically significant difference in the proportions of children aged less than 12 months compared with those aged 12–24 months, with the necrosis group having more patients aged < 12 months; however, no significant differences were found in other age groups.

**Table 1:** Comparison of general clinical data between intestinal necrosis group and control group

variable	Control group (*n* = 64)	Intestinal necrosis group (*n* = 23)	statistic	*P*
age (m)/n(%)			10.442^a^	0.005^*^
< 12	12^m^ (18.75)	12^m^ (52.17)		
12–24	24^n^ (37.50)	3^n^(13.04)		
> 24	28^m, n^(43.75)	8^m, n^ (34.78)		
gender/n(%)			1.448^a^	0.229
Man	45 (70.31)	13 (56.52)		
femal	19 (29.69)	10 (43.48)		
disease course (h)			583.500^b^	0.142
median(IQR)	12.00(6.00,15.00)	12.00 (6.50,50.00)		
abdominal pain or crying/n(%)			0.002^a^	0.963
No	2 (3.12)	0 (0.00)		
Yes	62 (96.88)	23 (100.00)		
vomit/n(%)			2.370^a^	0.124
No	37 (57.81)	9 (39.13)		
Yes	27 (42.19)	14 (60.87)		
Bloody stool/n(%)			10.649^a^	0.001^*^
No	53 (82.81)	11 (47.83)		
Yes	11 (17.19)	12 (52.17)		

Note: IQR: Interquartile spacing. SD: standard deviation. a: Chi-square test statistic *χ*² value. b: U value of Mann-Whitney test statistic. *: *P* < 0.05. m, n: comparison between groups The same letter indicates that there is no significant difference between groups, different letters indicate that there is a significant difference

**Table 2:** Comparison of ultrasonic index data between intestinal necrosis group and control group

variable	Control group (*n* = 64)	Intestinal necrosis group (*n* = 23)	statistic	*P*
position/n(%)			4.660^a^	0.097
right midabdomen	50 (78.12)	17 (73.91)		
right upper abdomen	13 (20.31)	3 (13.04)		
else	1 (1.56)	3 (13.04)		
length of the intussuscepted segment(mm)			521.000^b^	0.038^*^
Median (IQR)	57.50(53.00,66.25)	65.00 (54,98.50)		
concentric ring thickness (mm)			431.500^b^	0.003^*^
Median (IQR)	27.00(25.00,31.00)	30.00(28.00,33.00)		
concentric ring width (mm)			612.000^b^	0.232
Median (IQR)	33.00 (30.00,37.00)	34.00(31.00,38.00)		
concentric ring thickness to width ratio			554.000^b^	0.080
Median (IQR)	0.85(0.77,0.91)	0.88(0.85,0.93)		
maximum intussusception head diameters (mm)			668.000^b^	0.512
Median (IQR)	14.00 (12.00,15.70)	14.20(12.00,16.50)		
maximum intussusception neck diameters(mm)			616.000^b^	0.247
Median (IQR)	10.25 (8.67,12.05)	9.00 (8.00,11.75)		
head-to-neck diameter ratio			485.500^b^	0.016^*^
Median (IQR)	1.25 (1.15,1.46)	1.46 (1.25,1.58)		
maximum intussusception head to concentric ring thickness ratio			1.173^a^	0.244
Mean(SD)	0.50 ± 0.12	0.47 ± 0.09		
fat core thickness (mm)			602.500^b^	0.198
Median (IQR)	16.00 (13.00,18.00)	15.00(13.00,16.00)		
bowel wall thickness (mm)			482.500^b^	0.014^*^
Median (IQR)	6.00 (5.57,7.00)	7.20 (6.20,8.10)		
peritoneal effusion/n(%)			6.412^a^	0.011^*^
no	51 (79.69)	12 (52.17)		
yes	13 (20.31)	11 (47.83)		
mesenteric lymphadenopathy/n(%)			1.248^a^	0.264
no	36 (56.25)	16 (69.57)		
yue	28 (43.75)	7 (30.43)		
blood flow signal/n(%)			20.407^a^	< 0.001^*^
Grade 1–2	3 (4.69)	5 (21.74)		
Grade 3	11 (17.19)	12 (52.17)		
Grade 4	50 (78.12)	6 (26.09)		
roundness			380.000^b^	0.001^*^
Median (IQR)	0.90 (0.87,0.93)	0.94 (0.92,0.96)		

Note: IQR: Interquartile spacing. SD: standard deviation. a: Chi-square test statistic *χ*² value. b: U value of Mann-Whitney test statistic. *: *P* < 0.05

**Table 3:** Comparison of biochemical analysis data between intestinal necrosis group and control group

variable	Control group (*n* = 64)	Intestinal necrosis group (*n* = 23)	statistic	*P*
CRP(mg/L)			548.000^b^	0.071
Median (IQR)	4.55(2.15,9.27)	1.60 (0.90,7.70)		
NLR			398.500^b^	0.001^*^
Median (IQR)	1.54 (0.86,2.68)	2.96 (1.52,4.04)		

Note: IQR: Interquartile spacing. b: U value of Mann-Whitney test statistic. *: *P* < 0.05

#### Multivariate analysis of clinical data in the bowel necrosis group

Variables with statistically significant differences (*P* < 0.05) between the necrosis and control groups in univariate analysis were included in the multivariate regression model. The final multivariate model included the following variables: age, roundness, concentric ring thickness, intussuscepted segment length, head-to-neck diameter ratio, bowel wall thickness, blood flow signal grading, NLR, peritoneal effusion, and bloody stools. A stepwise regression method was used to select variables, given the events-per-variable ratio. Roundness was multiplied by 100 to address its small individual differences, and the ROC curve analysis showed that optimal cutoff value of roundness when the Youden index reaches its Maximum was 91.75.Blood flow signal grades 1–2 were combined with grade 3 to compare against grade 4 due to a limited sample size. The results indicated that roundness (scaled by 100) and blood flow signal grading were independent predictors of bowel necrosis (*P* < 0.05) (Table 4).

**Table 4:** Logistic regression analysis of intestinal necrosis caused by intussusception in children

variable	β	OR (95%CI)	*P*
age(take > 24 m as reference)			0.075
< 12 m	0.553	1.739 (0.316, 9.578)	0.525
12 ~ 24 m	−2.913	0.054 (0.003, 1.058)	0.055
roundness(×100)	0.334	1.397 (1.086, 1.796)	0.009^*^
length of the intussuscepted segment	0.044	1.045 (0.999, 1.093)	0.056
blood flow signal grade(take grade 4 as reference)	2.308	10.051 (1.843, 54.807)	0.008^*^
peritoneal effusion	1.669	5.308 (0.916, 30.757)	0.063
bloody stool	1.562	4.766 (0.807, 28.144)	0.085

Note: *OR*: odds ratio. *CI*: confidence interval. *: *P* < 0.05

Receiver operating characteristic curve analysis for the intussusception bowel necrosis patients and discriminative ability of the bowel necrosis model.

The final model included roundness and blood flow signal grading as variables, with roundness treated as a continuous variable in the predictive model for bowel necrosis in intussusception, expressed as: In $$\left(P/1-P\right)=-25.140+0.277\times\mathrm{roundness}\times100+$$$$2.397\times\mathrm{blood}\;\mathrm{flow}\;\mathrm{signal}\;\mathrm{grade}$$ where *P* represents the probability of bowel necrosis in intussusception. A blood flow signal grade of 4 was coded as 0, whereas other grades were coded as 1 (Table 5).

**Table 5:** Predictive model parameters of intestinal necrosis due to intussusception in children

variable	β	OR (95%CI)	*P*
roundness(×100)	0.277	1.319 (1.095, 1.588)	0.004^*^
blood flow signal grade(take grade 4 as reference)	2.397	10.992 (3.250, 37.175)	< 0.001^*^

Note: *OR*: odds ratio. *CI*: confidence interval. *: *P* < 0.05

**Fig. 3:**
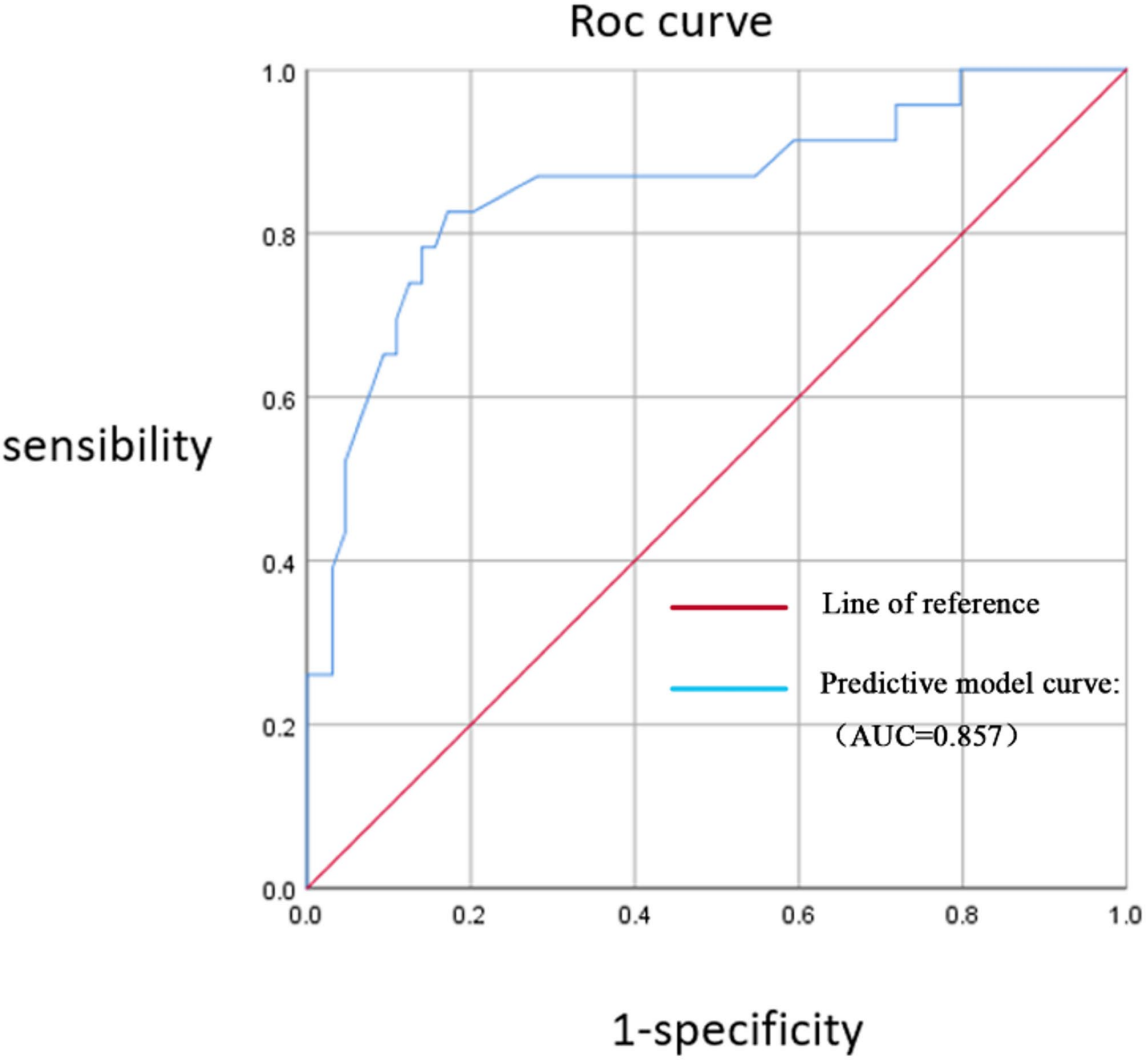
Receiver operating characteristic curve analysis of intussusception children combined with roundness and flow signal classification

ROC curves were generated for the probabilities of bowel necrosis based on roundness alone, blood flow signal grading alone, and the combined model to evaluate the predictive accuracy of the model. Using roundness alone yielded an AUC of 0.742 (95% *CI*: 0.630–0.854). Using blood flow signal grading alone yielded an AUC of 0.771 (95% *CI*: 0.652–0.891). The combined model, incorporating both roundness and blood flow signal grading, achieved an AUC of 0.857 (95% *CI*: 0.757–0.957), indicating good discriminative ability (Fig. 3).

## Discussion

If not promptly reduced, intussusception can lead to bowel obstruction, necrosis, and life-threatening situations in children [17]. Whether intussusception progresses to necrosis and whether and whether conservative or surgical intervention is required remain critical concerns in pediatric care.

Necrosis is a severe complication of intussusception, with studies demonstrating that younger age correlates with higher risk [9, 18]. Huang et al. [9] retrospectively analyzed 728 cases of intussusception where air enema reduction failed, comparing necrosis and non-necrosis groups. They found that the median age of participants in the necrosis group was significantly lower than that in the non-necrosis group (5.4 vs. 7.2 months, *P* = 0.000).Our study similarly found a higher proportion of necrosis cases in children aged < 12 months. Possible reasons likey were as follows: (1) younger children have narrower bowel lumina, thinner intestinal walls and immature vascular function, increasing susceptibility to ischemic necrosis; (2) younger children often can not articulate symptoms clearly, delaying diagnosis and raising the the risk of complications [19]; and (3) younger children have a more mobile ileocecal mesentery, which is less securely fixed, predisposing them to complex intussusception. Complex intussusception involves more bowel wall layers within a single intussuscepted segment, leading to more severe edema and pathological changes in the intussusception head. This increased tension on the bowel and mesentery raises the likelihood of necrosis.In this study, children aged > 24 months in the necrosis group (34.78%) did not significantly differ from those aged < 12 months. Pathological lead points triggering necrosis, such as inverted Meckel’s diverticulum or appendicitis, may be more common in children aged > 24 months [20, 21]. The pathological results showed that six of eight (75%) children in the necrosis group aged > 24 months had pathologies such as polyps, lymphoma, intestinal duplication, or ectopic pancreas. When a pathological lead point is present within the intussusception, it creates more content in the intussuscepted segment, compressing the mesentery and increasing the risk of bowel ischemia and infarction due to vascular twisting or severe constriction.

Peritoneal effusion may serve as an indirect sign of ischemic bowel necrosis in intussusception, typically manifesting as blood-tinged fluid, potentially due to mucosal secretion into the peritoneal cavity from ischemic necrosis in the bowel [8]. Chen et al. [8] found peritoneal effusion in all 48 cases of intussusception with bowel necrosis in their study. Zhou et al. [22] similarly noted peritoneal effusion accumulation in incarcerated hernia combined with intestinal necrosis was significantly higher than that in control group. Yu et al. [23] conducted a study on 547 cases of failed air enema reduction. Through forward stepwise regression, four variables - symptom duration, C-reactive protein, white blood cells, and ascites - were selected to be included in the nomogram. The consistency index was 0.871 (95% confidence interval: 0.834–0.908).Cases were randomly split into training (411 cases) and validation (136 cases) datasets, revealing no significant difference in effusion rates between the 2 groups (44.3% vs. 44.9%, *P* = 0.9868). Our study showed a higher proportion of peritoneal effusion in the necrosis group than in the control group (47.8% vs. 20.3%, *P* < 0.05).

Bloody stools are a common clinical sign of intussusception and suggest compromised blood flow to the bowel, with possible ischemic necrosis of some mucosal segments and impaired vascular function [24–26]. Rayamajhi et al. [27] identified bloody stools as an independent predictor of bowel necrosis. Although the necrosis group in this study had a higher incidence of bloody stools than the control group (52.17% vs. 17.19%, *P* < 0.05), bloody stools were not statistically significant in multivariate analysis (*P* = 0.085). This might be due to the emphasis of the multivariate model on an optimal predictor combination to achieve maximal predictive power.

Guo et al. [28] analyzed 190 cases of pediatric intussusception and found that radiological findings were shown to have a high concordance with pathology in the assessment of intussusception. For intussusception intestinal necrosis, the signs on plain films were clearer than those on colour Doppler sonography. However, Lagalla et al. [29] found that bowel necrosis presented as absent or reduced blood flow signal on CDFI. Lam et al. [30] reported when sonographic features of loss of blood fiow to the intussusception are present in latevintussusception, surgical intervention is required. These suggest that reduced or absent blood flow may predict bowel necrosis. CDFI blood flow provides diagnostic and therapeutic guidance. In our study, the intussusception flow signal was graded.The Limberg semi-quantitative method [13] was used to grade blood flow at the neck of the intussuscepted segment, with 73.91% of necrosis cases showing grades < 4. Compared with the control group, fewer patients in the necrosis group showed grade 4 blood flow (26.09% vs. 78.12%, *P* < 0.05), suggesting a significant reduction in blood flow signals in cases of bowel necrosis. Multivariate analysis confirmed blood flow grading as an independent predictor of bowel necrosis, indicating that low-grade blood flow signals (< grade 4) should caution against blindly using enema reduction in such patients.

This study showed that the necrosis group had greater concentric ring thickness and bowel wall thickness due to increased intussuscepted contents compressing the bowel wall, impairing blood supply, and causing congestion [8]. The necrosis group also showed a longer intussuscepted segment than the control group, potentially due to longer segments requiring more time and effort to reduce, thereby increasing the risk of bowel necrosis [31]. A larger head-to-neck diameter ratio in the necrosis group was also correlated with a higher risk of bowel necrosis, presumably because the edematous intussusception head and narrow neck hindered reduction, leading to necrosis.

Yoon et al. [10, 32] used roundness to assess breast tumor shape, noting that benign-appearing small triple-negative breast cancers and fibroadenomas had clear margins and parallel growth patterns. However, triple-negative cancers tended to be more invasive and proliferate rapidly often appearing rounder early on. Their results showed that triple-negative tumors had a higher mean roundness than fibroadenomas (60 ± 12% vs. 53 ± 13%, *P* < 0.05) [32]. Based on our finding that the necrosis group had greater concentric ring thickness, we hypothesized that the cross-sections in the necrosis group might be closer to circular. The median concentric ring thickness-to-width ratio showed an upward trend in both groups, but without statistical significance. However, the median roundness was higher in the necrosis group than in the control group, indicating that a more circular cross-section may increase the risk of bowel necrosis in intussusception.

This study has some limitations. First, the study was a retrospective analysis, so the results may be affected by information bias or selection bias. Second, this was a single-center study, so the generalizability of the research results cannot be confirmed. Third, since the number of patients with intestinal obstruction causing intestinal necrosis was very small (due to the low risk of intestinal necrosis), this study may not be sufficient to discover some true differences between the intestinal necrosis group and the non-intestinal necrosis group. Fourth, other unknown factors not included in the analysis may also have an impact on the results. Large-scale, prospective, multi-center studies are needed to further evaluate the relevant factors of intestinal necrosis in intussusception.

## Conclusions

Our study indicated that low CDFI grading and high roundness values were associated with an increased risk of bowel necrosis in intussusception. Therefore, we recommend that aggressive surgical intervention should be considered over routine enema reduction for pediatric intussusception cases with a high suspicion of bowel necrosis.

## Supplementary Information

Supplementary material 1. Additional file1. Fig. 1 Intussusception. Male child, 14 months. a. The cross section of intussusception lesions showed a"concentric circle sign" (red arrow). b. The longitudinal section of intussusception lesions showed "false kidney sign" (red outline). Additional file 2. Fig. 2 Transverse view of intussusception. Male child, 14 months. Arrow A refers to the outer intestinal tract of intussusception (the part between the red circle and the yellow circle), arrow B refers to the inserted intestinal tract (the part inside the yellow circle), arrow C refers to the hyperechoic fat, arrow D refers to the hypoechoic lymph node; EF is the thickness of "concentric ring" and GH is the width of"concentric ring".

Supplementary material 2. Additional file 3. Longitudinal view of intussusception. Male child, 14 months. The red dashed line indicated by D1 is maximum diameter of intussusception head, the red dashed line indicated by D2 is maximum diameter of intussusception neck, and the red line indicated by L is the length of the intussusception. Additional file 4. Fig. 4 Diagram of lymph node measurement. Male child, 14 months. AB is the long diameter of lymph node, CD is the short diameter of lymph node

## Data Availability

Data is provided within the manuscript or supplementary information files.

## References

[1] Applegate KE. Intussusception in children: evidence-based diagnosis and treatment. Pediatr Radiol. 2009;39(Suppl 2):S140–3. 10.1007/s00247-009-1178-9 19308373

[2] Marsicovetere P, Ivatury SJ, White B, Holubar SD. Intestinal intussusception: etiology, diagnosis, and treatment. Clin Colon Rectal Surg. 2017;30:30–9. 28144210 10.1055/s-0036-1593429 PMC5179276

[3] Mandeville K, Chien M, Willyerd FA, Mandell G, Hostetler MA, Bulloch B. Intussusception: clinical presentations and imaging characteristics. Pediatr Emerg Care. 2012;28:842–4. 22929138 10.1097/PEC.0b013e318267a75e

[4] Kaplan SL, Magill D, Felice MA, Edgar JC, Anupindi SA, Zhu X. Intussusception reduction: effect of air vs. liquid enema on radiation dose. Pediatr Radiol. 2017;47:1471–6. 28578475 10.1007/s00247-017-3902-1

[5] Daneman A, Navarro O. Intussusception. Part 2: an update on the evolution of management. Pediatr Radiol. 2004;34:97–108. 14634696 10.1007/s00247-003-1082-7

[6] Demir IE, Ceyhan GO, Friess H. Beyond lactate: is there a role for serum lactate measurement in diagnosing acute mesenteric ischemia? Dig Surg. 2012;29:226–35. 22699523 10.1159/000338086

[7] Hanquinet S, Anooshiravani M, Vunda A, Le Coultre C, Bugmann P. Reliability of color doppler and power doppler sonography in the evaluation of intussuscepted bowel viability. Pediatr Surg Int. 1998;13(5–6):360–2. 9639617 10.1007/s003830050339

[8] Chen WJ, Zhang HR, Liu JQ, Hu Y, Chen J, Yang F. [Ultrasonographic findings of intussusception complicated by intestinal necrosis in children]. Zhongguo Dang Dai Er Ke Za Zhi. 2008;10:161–2. Chinese. 18433537

[9] Huang HY, Huang XZ, Han YJ, Zhu LB, Huang KY, Lin J, et al. Risk factors associated with intestinal necrosis in children with failed non-surgical reduction for intussusception. Pediatr Surg Int. 2017;33:575–80. 28124113 10.1007/s00383-017-4060-0

[10] Bae MS, Seo M, Kim KG, Park IA, Moon WK. Quantitative MRI morphology of invasive breast cancer: correlation with immunohistochemical biomarkers and subtypes. Acta Radiol. 2015;56:269–75. 24558165 10.1177/0284185114524197

[11] Zhang Y, Shao CC, Wei XL, Ni PJ, Guan H, Zhao C. etal. Ultrasound findings to predict risk of recurrence in pediatric intussusception after air enema reduction. J Ultrasound Med. 2022;41:1227–35. 34418137 10.1002/jum.15814

[12] Zhang J, Dong Q, Su X, Long J. Factors associated with in-hospital recurrence of intestinal intussusception in children. BMC Pediatr. 2023;23:428. 37633888 10.1186/s12887-023-04267-9 PMC10464288

[13] Cleveland NK, Picker EA, Dolinger MT, Rubin DT. The arrival of intestinal ultrasound for inflammatory bowel disease care in the united States. Gastroenterol Hepatol (N Y). 2023;19:147–54. 37706105 PMC10496276

[14] Tseng HS, Wu HK, Chen ST, Kuo SJ, Huang YL, Chen DR. Speckle reduction imaging of breast ultrasound does not improve the diagnostic performance of morphology-based CAD system. J Clin Ultrasound. 2012;40:1–6. 22086841 10.1002/jcu.20897

[15] Ma GMY, Lillehei C, Callahan MJ. Air contrast enema reduction of single and recurrent Ileocolic intussusceptions in children: patterns, management and outcomes. Pediatr Radiol. 2020;50:664–72. 32006065 10.1007/s00247-020-04612-5

[16] Kelley-Quon LI, Arthur LG, Williams RF, Goldin AB, St Peter SD, Beres AL, et al. Management of intussusception in children: A systematic review. J Pediatr Surg. 2021;56:587–96. 33158508 10.1016/j.jpedsurg.2020.09.055 PMC7920908

[17] Fan WF, Ma G, Li GC, Long J, Xu YH, Guo KJ, et al. Ileocecal intussusception caused by two different tumors - which is the culprit lesion? A case report. World J Clin Cases. 2020;8:2044–49. 32518799 10.12998/wjcc.v8.i10.2044 PMC7262695

[18] Chen SC, Wang JD, Hsu HY, Leong MM, Tok TS, Chin YY. Epidemiology of childhood intussusception and determinants of recurrence and operation: analysis of National health insurance data between 1998 and 2007 in Taiwan. Pediatr Neonatol. 2010;51:285–91. 20951359 10.1016/S1875-9572(10)60055-1

[19] Hu J, Liu M, Yu X, Xia Q, Wang K, Guo S, et al. Clinical characteristics of intussusception with surgical reduction: a Single-Center experience with 568 cases. J Gastrointest Surg. 2019;23:2255–62. 30859429 10.1007/s11605-019-04178-0

[20] St-Vil D, Brandt ML, Panic S, Bensoussan AL, Blanchard H. Meckel’s diverticulum in children: a 20-year review. J Pediatr Surg. 1991;26:1289–92. 1812259 10.1016/0022-3468(91)90601-o

[21] Staatz G, Alzen G, Heimann G. Darminfektion, die Häufigste invaginationsursache Im kindesalter: ergebnisse einer 10jährigen klinischen studie [Intestinal infection, the most frequent cause of invagination in childhood: results of a 10-year clinical study]. Klin Padiatr. 1998;210:61–4. 9561958 10.1055/s-2008-1043851

[22] Zhou J, Yuan X. Establishment of a risk prediction model for bowel necrosis in patients with incarcerated inguinal hernia. BMC Med Inf Decis Mak. 2024;24:39. 10.1186/s12911-024-02440-3 PMC10845797 38321399

[23] Yu YY, Zhang JJ, Xu YT, Lin ZX, Guo SK, Li ZR, et al. Developing and validating a nomogram for early predicting the need for intestinal resection in pediatric intussusception. Front Pediatr. 2024;12:1409046. 38774298 10.3389/fped.2024.1409046 PMC11106445

[24] Kishi Y, Kopetz S, Chun YS, Palavecino M, Abdalla EK, Vauthey JN. Blood neutrophil-to-lymphocyte ratio predicts survival in patients with colorectal liver metastases treated with systemic chemotherapy. Ann Surg Oncol. 2009;16:614–22. 19130139 10.1245/s10434-008-0267-6

[25] Koch J, Harder T, von Kries R, Wichmann O. Risk of intussusception after rotavirus vaccination. Dtsch Arztebl Int. 2017;114:255–62. 28468712 10.3238/arztebl.2017.0255 PMC5424085

[26] Kimia AA, Williams S, Hadar PN, Landschaft A, Porter J, Bachur RG. Positive guaiac and bloody stool are poor predictors of intussusception. Am J Emerg Med. 2018;36:931–4. 29079372 10.1016/j.ajem.2017.10.051

[27] Rayamajhi A, Thapa A, Kumar M, Yen C, Tate JE, Parashar UD, et al. Preparing for rotavirus vaccine introduction - A retrospective assessment of the epidemiology of intussusception in children below 2 years of age in Nepal. Vaccine. 2018;36:7836–40. 29169894 10.1016/j.vaccine.2017.11.022 PMC6534114

[28] Guo WL, Wang J, Zhou M, Sheng M, Fang L. The role of plain radiography in assessing intussusception with vascular compromise in children. Arch Med Sci. 2011;7:877–81. 22291835 10.5114/aoms.2011.25565 PMC3258819

[29] Lagalla R, Caruso G, Novara V, Derchi LE, Cardinale AE. Color doppler ultrasonography in pediatric intussusception. J Ultrasound Med. 1994;13:171–4. 7932972 10.7863/jum.1994.13.3.171

[30] Lam AH, Firman K. Value of sonography including color doppler in the diagnosis and management of long standing intussusception. Pediatr Radiol. 1992;22:112–4. 1501937 10.1007/BF02011308

[31] Khorana J, Singhavejsakul J, Ukarapol N, Laohapensang M, Siriwongmongkol J, Patumanond J. Prognostic indicators for failed nonsurgical reduction of intussusception. Ther Clin Risk Manag. 2016;12:1231–7. 27563245 10.2147/TCRM.S109785 PMC4984823

[32] Yoon GY, Cha JH, Kim HH, Shin HJ, Chae EY, Choi WJ. Sonographic features that can be used to differentiate between small triple-negative breast cancer and fibroadenoma. Ultrasonography. 2018;37:149–56. 28870060 10.14366/usg.17036 PMC5885477

