# Supplementary material for: Predicting bowel necrosis in pediatric acute intussusception using roundness and other related factors
Source: BMC Pediatr. 2025 Oct 21;25:843. doi: 10.1186/s12887-025-06172-9 (PMC12542266; doi:10.1186/s12887-025-06172-9)
Supplement: Supplementary file 2 — Supplementary material 2. Additional file 3. Longitudinal view of intussusception. Male child, 14 months. The red dashed line indicated by D1 is maximum diameter of intussusception head, the red dashed line indicated by D2 is maximum diameter of intussusception neck, and the red line indicated by L is the length of the intussusception. Additional file 4. Fig. 4 Diagram of lymph node measurement. Male child, 14 months. AB is the long diameter of lymph node, CD is the short diameter of lymph node [file 12887_2025_6172_MOESM2_ESM.pdf]

江南大学附属儿童医院
Affiliated Children's Hospital of Jiangnan University
伦理审查批件
IEC Approval Notice

批件号<br>Approval Number	WXCH2024-11-137		
项目名称<br>Protocol title	Predicting Bowel Necrosis in Pediatric Acute Intussusception Using Roundness and Other Related Factors<br>圆度等相关因素预测儿童急性肠套叠发生肠坏死的价值分析		
项目来源<br>Source	无<br>None		
研究单位<br>Study site	江南大学附属儿童医院<br>Affiliated Children's Hospital of Jiangnan University		
主要研究者<br>Principal Investigtor	徐欣欣<br>Xu Xinxin		
审查类别<br>Type of review	初始审查<br>Initial review	审查方式<br>Type of review	快速审查<br>Expedited review
审查日期<br>Date	2024/11/25	审查地点<br>Place	网络审查<br>Network review
审阅文件<br>Submission Documents	1．研究方案（版本号：V1.0，版本日期：2024/11/18）<br>2．知情同意书（版本号：V1.0，版本日期：2024/11/18）		

审查意见 Evaluation Comments

■同意<br>Approval	□作必要修正后同意<br>Minor revised	□作必要修正后重审<br>Major revised	□不同意<br>Disapproval

意见说明 Comments Description

1. 根据国家相关法规，本伦理委员会的组织和实施相对独立。

According to relevant national regulations, the organization and implementation of this ethics committee are relatively independent.

2. 本伦理委员会的人员组成和工作程序是国家相关规定，符合《赫尔辛基宣言》的准则。

The composition and working procedures of this ethics committee are in accordance with relevant national regulations and the guidelines of the Helsinki Declaration.

3. 本项目所有研究参与者均需获得参与知情同意，遵循伦理委员会批准的方案开展临床研究，保护受试者的健康与权利。

All research participants in this project must obtain informed consent and follow the approved protocol of the ethics committee to conduct clinical research and protect the health and rights of the subjects.

江南大学附属儿童医院伦理委员会

2024 年 11 月 26 日

（印章：江南大学附属儿童医院伦理委员会）

江南大学附属儿童医院伦理委员会　地址：无锡市清扬路 299-1 号　邮编：214023
办公室电话：0510-85350613　传真：0510-85350738　E-mail: etyyyjb@163.com
